# Neurons are MHC Class I-Dependent Targets for CD8 T Cells upon Neurotropic Viral Infection

**DOI:** 10.1371/journal.ppat.1002393

**Published:** 2011-11-17

**Authors:** Grégoire Chevalier, Elsa Suberbielle, Céline Monnet, Valérie Duplan, Guillaume Martin-Blondel, Fanny Farrugia, Gwendal Le Masson, Roland Liblau, Daniel Gonzalez-Dunia

**Affiliations:** 1 Inserm, U1043, Toulouse, France; 2 CNRS, U5282, Toulouse, France; 3 Université de Toulouse, UPS, Centre de Physiopathologie de Toulouse Purpan (CPTP), Toulouse, France; 4 INSERM, U862 and Université Bordeaux 2, Bordeaux, France; The Fox Chase Cancer Center, United States of America

## Abstract

Following infection of the central nervous system (CNS), the immune system is faced with the challenge of eliminating the pathogen without causing significant damage to neurons, which have limited capacities of renewal. In particular, it was thought that neurons were protected from direct attack by cytotoxic T lymphocytes (CTL) because they do not express major histocompatibility class I (MHC I) molecules, at least at steady state. To date, most of our current knowledge on the specifics of neuron-CTL interaction is based on studies artificially inducing MHC I expression on neurons, loading them with exogenous peptide and applying CTL clones or lines often differentiated in culture. Thus, much remains to be uncovered regarding the modalities of the interaction between infected neurons and antiviral CD8 T cells in the course of a natural disease. Here, we used the model of neuroinflammation caused by neurotropic Borna disease virus (BDV), in which virus-specific CTL have been demonstrated as the main immune effectors triggering disease. We tested the pathogenic properties of brain-isolated CD8 T cells against pure neuronal cultures infected with BDV. We observed that BDV infection of cortical neurons triggered a significant up regulation of MHC I molecules, rendering them susceptible to recognition by antiviral CTL, freshly isolated from the brains of acutely infected rats. Using real-time imaging, we analyzed the spatio-temporal relationships between neurons and CTL. Brain-isolated CTL exhibited a reduced mobility and established stable contacts with BDV-infected neurons, in an antigen- and MHC-dependent manner. This interaction induced rapid morphological changes of the neurons, without immediate killing or impairment of electrical activity. Early signs of neuronal apoptosis were detected only hours after this initial contact. Thus, our results show that infected neurons can be recognized efficiently by brain-isolated antiviral CD8 T cells and uncover the unusual modalities of CTL-induced neuronal damage.

## Author Summary

When a virus infects the brain, it is important to quickly block viral replication without causing excessive damage to neurons, which are not easily renewed. Cytotoxic T lymphocytes (CTL) are one of the main actors for virus elimination. However, the question of whether CTL are indeed capable of destroying infected neurons remains controversial. For this work, we analyzed the characteristics of interactions between infected neurons and CTL using neurotropic Borna disease virus (BDV). This virus infects neurons and triggers severe inflammation in the brain. We isolated CTL directly from the brains of rats infected with BDV and analyzed their interaction with primary cultures of neurons. Using live-cell fluorescence microscopy, we observed that CTL were arrested upon encounter with infected neurons and that they established stable contacts with them. Thereafter, infected neurons exhibited rapid changes in permeability but remained alive and electrically active for several hours, before ultimately being destroyed. Our study shows that neurons can indeed be recognized by CTL, an important observation for a better understanding of the physiopathology of virus-induced brain inflammation. In addition, it reveals that neurons are relatively resistant to CTL-induced killing, which may open a window of opportunity for new treatments.

## Introduction

A better understanding of the interactions between viruses and the central nervous system (CNS) represents a major issue in viral pathogenesis. Indeed, viral persistence in the CNS represents a challenge both for the host and the pathogen. On the virus side, it is essential to adapt a strategy of replication that will minimize virus-induced cell damage and limit its recognition by the immune response. On the host side, it is essential to quickly halt virus multiplication, while causing minimal damage to CNS resident cells and in particular to neurons which have limited capacities of renewal [1], [2]. These issues are complicated by the unique immunologic properties of the CNS, originally referred to as an immune privileged site. It is now clear that this “privilege” is very relative and that despite the blood-brain barrier and the absence of dedicated lymphoid drainage [3], the immune response can generally control invasion of the CNS by pathogens, although often at the expense of irremediable tissue damage due to excessive inflammation.

Among the different immune effectors involved in viral elimination, CD8 T cells have received much attention, owing to their essential roles in the primary protection of the host against infectious diseases. CD8 cytotoxic T lymphocytes (CTL) mediate their antiviral effects by recognizing viral peptides presented by class I major histocompatibility complex (MHC I) molecules. Upon engagement of the T cell receptor (TCR) with the peptide-MHC I complex, CTL mediate cell killing essentially through two independent pathways: perforin-dependent delivery of granzymes and interaction of Fas-ligand (FasL) with the Fas-receptor on the target cell surface. These lytic mechanisms could, however, have devastating consequences in the CNS and it has been shown that alternative non-cytolytic mechanisms can also be engaged by CTL [4], [5]. In particular, antiviral cytokines produced by CD8 T cells, such as interferon gamma (IFN-γ) or tumor necrosis factor alpha (TNF-α), can stimulate intracellular pathways that interfere with viral replication, resulting in complete or partial clearance from the cell without destroying it. Furthermore, both mechanisms can be combined to limit viral multiplication in the brain. One example is infection with Herpes simplex virus type 1 (HSV-1), for which latency is controlled by IFN-γ produced by CD8 T and by granzyme B-mediated degradation of the HSV-1 immediate early protein ICP4 [6], [7].

As recognition of peptide-MHC I complexes is essential to elicit CTL effector functions, it has long been considered that neurons were spared from CD8 T cell attack because they did not express MHC I molecules [8], [9]. Recent studies, however, have challenged this view. First, accumulating evidence has revealed that MHC I expression in the developing and adult CNS can have additional non-immune functions. Indeed, neuronal MHC I molecules appear to be crucial for normal brain development, neuronal differentiation and plasticity [10], [11]. In addition to their role in CNS function, it was also shown that neurons could express MHC I molecules under inflammatory conditions. For example, in Rasmussen's encephalitis, histopathological examination of autopsy material revealed the presence of granzyme B-containing CD8 T cells in direct apposition to MHC I positive neurons [12]. Further evidence of MHC I inducibility in neurons was provided *in vitro*. Indeed, it has been shown that primary cultures of neurons treated with IFN-γ and electrically silenced using Tetrodotoxin (TTX) can be induced to express MHC I molecules on their surface [13]. This expression is functionally relevant, as CTL can attack peptide-pulsed neurons in an antigen-specific manner *in vitro* [14]. One caveat of such studies, however, is that they were performed using neurons loaded with non-limiting amounts of exogenous peptides. Moreover, CD8 T cells used in these assays were either CTL clones or lines with pre-defined epitope specificity, often derived for practical reasons from TCR-transgenic animals. CTL were also often further differentiated *in vitro* or restimulated in culture. Despite the above-mentioned limitations, several studies using variations of this experimental paradigm have provided considerable insight on MHC I-restricted T cell interactions with neurons [2], [15]. One still unsatisfactorily resolved issue in neurovirology, however, is whether antiviral CD8 T cells can indeed recognize and directly attack virus-infected neurons in the course of a natural disease.

Infection with Borna disease virus (BDV) appears as a very suitable model system to address these questions. BDV is an enveloped virus with a non-segmented, negative strand RNA genome [16], [17], which is characterized by a remarkable non-cytolytic strategy of replication. BDV infects the CNS of a wide variety of mammals [18], [19] and induces a large spectrum of neurological disorders [18], [20], [21]. One of the best-investigated animal models for the pathogenesis of BDV infection is the Lewis rat. After intracerebral infection, the rats develop an acute meningo-encephalitis in which the infiltrating immune cells are mainly comprised of CD8 T cells, together with less numerous CD4^+^ T cells and macrophages [22], [23]. Several studies showing the preferential presence of CD8 T cells in direct proximity to neuronal cell lesions and adoptive transfer experiments have clearly established the central role of CD8 T cells in BDV-induced encephalitis and neuronal destruction [24]–[26].

In this study, our goal was to provide further insight on the specifics on neuron / CD8 T cell interactions in the course of a natural disease and to determine the ensuing functional consequences. We used BDV-infected primary cultures of neurons, which were incubated, without any further manipulation, with CD8 T cells directly extracted from the brains of BDV-infected Lewis rats. Thereafter, we analyzed the outcome of the cognate interactions between CD8 T cells and BDV-infected neurons.

## Results

### BDV infection triggers neuronal MHC I expression

**Figure 1:**
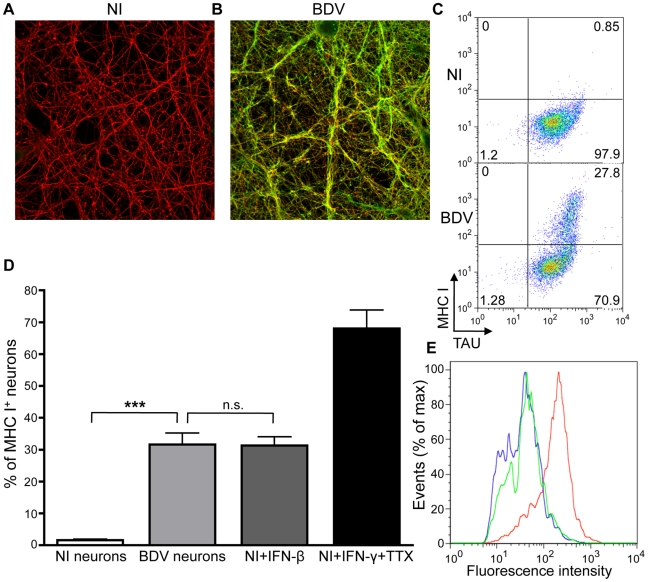
BDV infection induces MHC I expression on neurons. (A), Immunofluorescence analysis of non-infected (NI) and (B), BDV-infected neurons, 14 days post infection with cell-free BDV. BDV Nucleoprotein was detected using a rabbit polyclonal antibody, followed by an Alexa 488-coupled secondary antibody (green), while ßIII-tubulin neuronal protein was detected using a mouse monoclonal antibody, followed by an Alexa 594-coupled secondary antibody (red). (C) Representative dot plot examples of MHC I and neuron-specific Tau expression in non-infected (NI) or BDV-infected neurons. (D) Quantification of MHC I positive neurons. Data are represented as percentage of Tau^+^ neurons expressing MHC I. Results are expressed as means ± sem of at least four independent experiments. ***, p<0.001 by Mann-Whitney *U*-test. (E) Representative fluorescence intensity profiles for MHC I expression between BDV-infected neurons (green) and non-infected neurons treated with either IFN-β (blue) or IFN-γ+TTX (red).

We used cortical neurons prepared from Lewis rat embryos that had been infected with cell-free BDV one day after plating and further cultured for 14 days. At this time point, neurons have established mature synapses [27] and the virus has spread to all of them, in agreement with our previous findings [28]–[30]. Detection of the BDV nucleoprotein using immunofluorescence analysis (Figure 1A and B) confirmed that the large majority (>95%) of neurons was positive for BDV antigens and that infection proceeded without detectable effect on the morphology or viability of the cultures, consistent with the non-cytolytic replication strategy of BDV.

We then assessed expression of MHC I molecules on the neuronal surface, since this is a key prerequisite for antigen presentation to CD8 T lymphocytes. We used infected and non-infected neurons, as well as non-infected neurons that had been treated for 72 h with IFN-γ (100 U/ml) and TTX (1 µM), to induce MHC I expression [13]. We also used non-infected neurons treated for 72 h with IFN-ß (100 U/ml). Living neurons still attached to the plastic were stained using a monoclonal antibody recognizing the rat MHC I RT1-A molecule. After staining, neurons were rapidly harvested, fixed and further stained for the intracellular neuronal protein Tau. Flow cytometry analysis (Figure 1C and D) revealed that cultures contained >98% neurons and that non-infected neurons expressed little or no MHC I molecules (0.5±0.3% positive neurons). As previously published [13], treatment with IFN-γ and TTX strongly induced MHC I expression (70±5% positive neurons). Interestingly, BDV infection triggered a significant expression of neuronal MHC I (30±3.5% positive neurons). Analysis of fluorescence intensities (Figure 1E) revealed that levels of surface expression of MHC I induced by BDV infection were nevertheless lower than those obtained by neuronal exposure to IFN-γ and TTX. Of note, treatment of non-infected neurons with IFN-ß induced comparable proportion (30±3%) and levels of MHC I expression (Figure 1E) than infection with BDV.

Thus, BDV infects neuronal cultures efficiently and triggers significant neuronal MHC I expression. For all subsequent studies, we used neurons that had been infected for the same length of time (14 days) and verified for each experiment that infection was indeed complete prior to any subsequent analysis.

### CD8 T cells preferentially infiltrate the CNS upon BDV infection and express cytotoxic effectors

**Figure 2:**
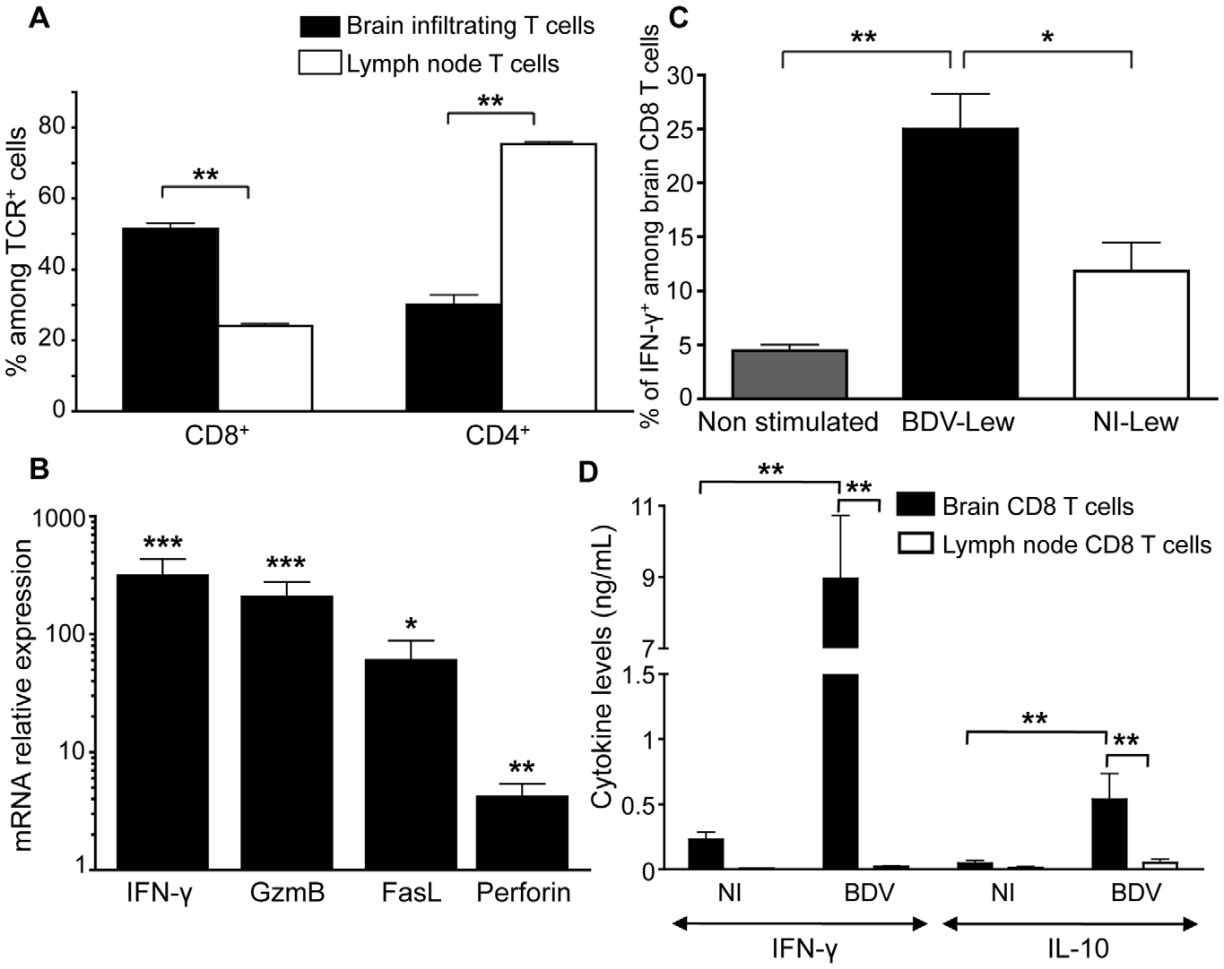
Characteristics of brain-purified CD8 T cells from BDV-infected rats. (A) Percentages of CD8^+^ versus CD4^+^ cells among TCRαβ^+^ cells purified from brain or cervical lymph nodes of infected rats. Values are expressed as means ± sem of six independent experiments **, p<0.01 by Mann-Whitney *U*-test. (B) Determination by real-time quantitative RT-PCR of the relative mRNA levels of IFN-γ, Granzyme B (GzmB), Fas-ligand (FasL) and Perforin in brain-purified CD8 T cells compared to cervical lymph node CD8 T cells of the same animal. Values were normalized for actin mRNA levels. Values are expressed as means ± sem of four independent experiments. ***, p<0.001; **, p<0.01; and *, p<0.05 by paired Student's *t*-test. (C) Percentage of IFN-γ-producing cells amongst TCRαβ^+^CD8^+^ cells after 48h restimulation with BDV-infected (BDV-Lew) or non-infected (NI-Lew) Lewis fibroblasts, as determined by intracellular FACS staining. Results are expressed as means ± sem of four independent experiments. *, p<0.05; **, p<0.01 by Mann-Whitney *U*-test. (D) Cytokine levels in supernatants of cocultures of CD8 T cells with neurons. Supernatants were assayed after 48 hours of culture using Luminex multiplex kits. Only levels of IFN-γ and IL-10 are shown (see Figure S1 for other cytokines). Values are expressed as mean concentrations ± sem of four independent experiments. **, p<0.01 by Mann-Whitney *U*-test.

Lewis rats were sacrificed 14 days after intracerebral infection with BDV. In previous experiments, we determined that, similar to others [24], [31], this time point corresponded to the peak of the neurological disease caused by BDV infection and that inflammatory infiltrates were most abundant in the brain at this stage [32]. Consistent with previous reports [24], [31], phenotypic characterization of brain-infiltrating lymphocytes using flow cytometry revealed that CD8 T cells accounted for 51%±1.5% of all T cells present in the brain (i.e., positive for the TCR), whereas CD4 T cells represented 30%±3% of TCR^+^ cells (Figure 2A). This 1.7 to 1 ratio of CD8 to CD4 cells in the brain contrasted with the usual 1 to 3 ratio found in cervical lymph nodes of the same animals, reflecting the high proportion of CD8 T cells recruited to the CNS upon BDV infection. Moreover, this ratio was probably under-estimated since we detected a high proportion of TCR^+^ cells (≈20%) that were negative for both CD4 and CD8 expression, presumably due to down-modulation of CD8 expression upon activation [33]. To further analyze the phenotype of CD8 T cells, we purified them using cell sorting (>99% pure), within a few hours after harvesting. We prepared total RNA from sorted CD8 T cells and analyzed expression of several genes by real-time quantitative RT-PCR. When compared to CD8 T cells purified from lymph nodes of the same animals (Figure 2B), brain-infiltrating lymphocytes expressed high levels of effector molecules such as IFN-γ (300-fold induction on average), Granzyme B (180-fold induction) or FasL (50-fold induction). Expression of Perforin mRNA was also significantly elevated, albeit at lower levels.

To determine the proportion of brain-derived CD8 T cells specific for BDV, highly purified CD8 T cells were stimulated with irradiated syngeneic Lewis fibroblasts (infected or not with BDV) and assayed by FACS for IFN-γ production. On average, 25%±3% of CD8 T cells were positive for IFN-γ following a 48 h-stimulation period, whereas this percentage was of 11%±2.5% upon culture with non-infected cells (Figure 2C).

Finally, we assessed whether brain- or lymph node-purified CD8 T cells secreted cytokines upon incubation with primary cultures of neurons that were infected with BDV or not. Cytokine levels in the supernatants were assayed at 48 h with a Luminex assay specific for 9 rat cytokines. We did not detect any cytokine above the threshold level of detection of the assay (4.88 pg/ml) in the supernatants of CD8 T cells purified from lymph nodes. In contrast, brain-purified CD8 T cells produced high levels of IFN-γ (up to 9 ng/ml) upon culture with infected neurons (Figure 2D). These cells also produced IL-10, although at levels below those of IFN-γ. Remarkably, levels were also much lower when brain-purified CD8 T cells were incubated with non-infected neurons, suggesting that cytokine secretion resulted mainly from the recognition of viral antigens presented by neurons. There were no significant differences regarding levels of IL-6, IL-17 or TNF-α (Figure S1), while results were below threshold for IL-4, IL-5, IL-9 and IL-13.

Together, these data show that BDV infection triggers a prominent recruitment of CD8 T cells in the brain. These cells are strongly activated, express high levels of effector molecules and produce large quantities of IFN-γ upon interaction with BDV-infected neurons.

**Figure 3:**
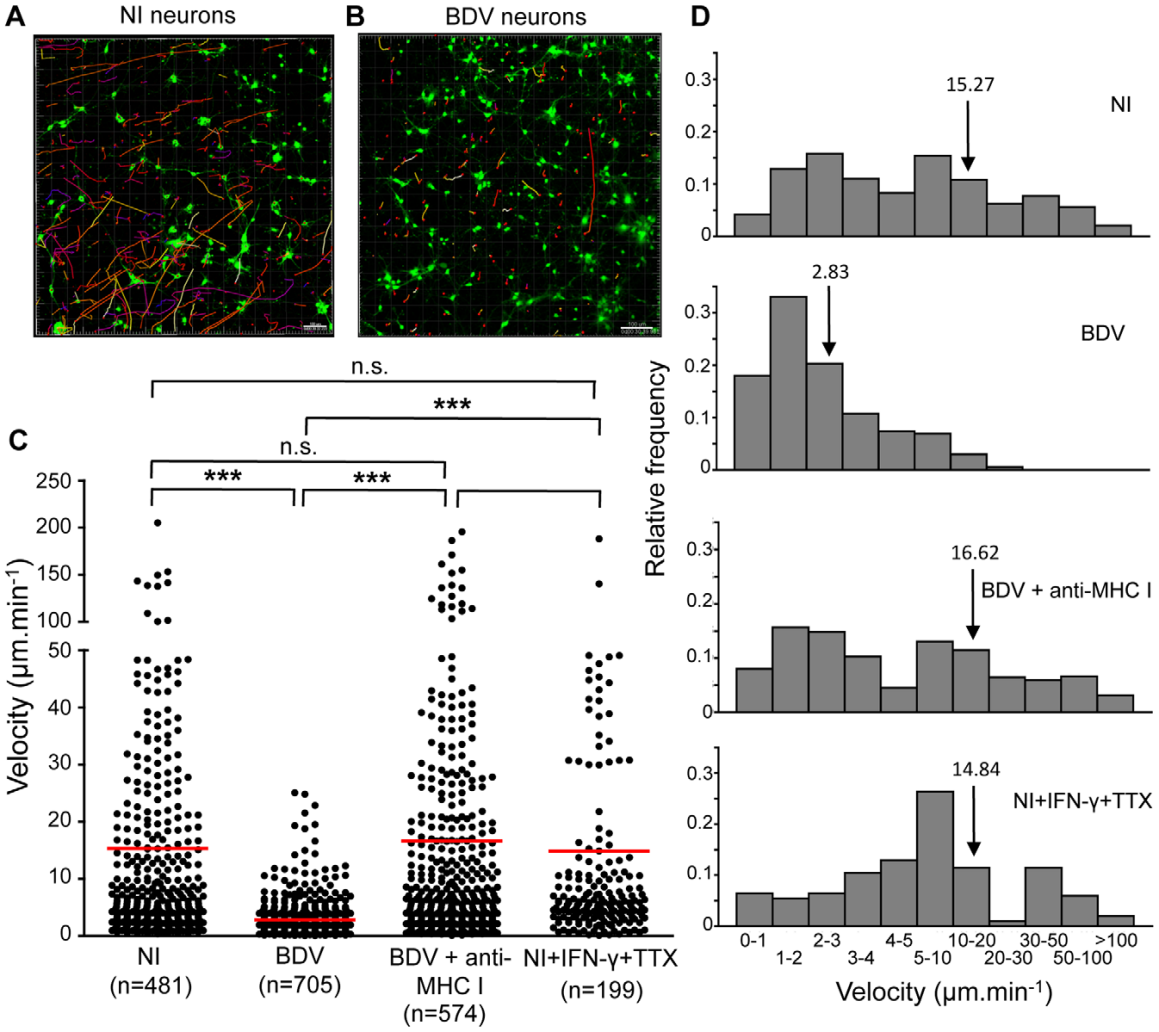
Quantitative analysis of CD8 T cell movement behavior in contact with neurons. CD8 T cells purified from the brain of BDV-infected rats were labeled with PKH-26 (red) and added to Calcein-AM labeled neurons (green) which were non-infected (A) or infected (B) with BDV. Randomly chosen trajectories of individual cells were automatically tracked using Imaris software. Trajectories are depicted as color-coded tracks to represent increasing time, from blue (start of imaging) to yellow (end of imaging). A representative imaging session of 15 min is shown in each case. (C), Determination of average CD8 T cell velocities for the different experimental conditions. Dots represent average cell velocities of individual cells (200 to 700 cells analyzed depending on the condition, see numbers below the graph), red bars indicate mean values. Analyses were performed using non-infected neurons (NI), non-infected neurons treated for 48 h with IFN-γ+TTX, BDV-infected neurons or BDV-infected neurons treated with a neutralizing MHC I monoclonal antibody 1 h prior to washes and addition of CD8 T cells. P values were calculated using Kruskal-Wallis test. ***, p<0.001; n.s., not significant. (D) Relative frequencies of brain CD8 T cells as a function of their individual mean velocity, under the different conditions of culture. Arrows point to the mean velocity for each condition.

Brain-purified CD8 T cells are arrested upon contact with infected neurons, which they recognize in an antigen- and MHC I-dependent manner. To get further insight on the dynamics of interactions between CD8 T cells and neurons, we stained purified CD8 T cells with the lipophilic dye PKH-26, added them to neuronal cultures previously labeled with Calcein-AM and performed confocal microscopy imaging using a temperature-controlled imaging setup (Figure 3, video S1 and video S2). Strikingly, the mobility of CD8 T cells isolated from the brain was extremely reduced upon incubation with BDV-infected neurons compared to non-infected ones (Figure 3A and 3B). Indeed, the measured mean velocity was 2.83 µm/min (95% confidence interval (CI) of mean 2.59 - 3.03) in the presence of infected neurons compared to 15.27 µm/min, (95% CI 13.00–17.53) with non-infected ones (Figure 3C and 3D). Furthermore, once arrested, CD8 T cells established stable interactions with BDV-infected neurons throughout the whole imaging period (up to 45 min). CD8 T cells were found in apposition to neurites and neuronal somas. In contrast, the same brain CD8 T cells exhibited a much more dynamic behavior upon incubation with non-infected neurons. Induction of MHC I expression at the surface of non-infected neurons with IFN-γ and TTX was not sufficient to arrest CD8 T cells (mean velocity 14.84 µm/min, 95% CI 11.60–18.07). Conversely, masking MHC I molecules on BDV-infected neurons using OX-18 antibody restored a high mobility of CD8 T cells (16.62 µm/min, 95% CI 14.24–19.00). In addition, relative frequencies of CD8 T cell moving at various velocities were similar when comparing non-infected neurons, non-infected neurons treated with IFN-γ and TTX or BDV-infected neurons treated with anti-MHC I antibody (Figure 3D). Finally, CD8 T cells purified from cervical lymph nodes of the same infected animals were highly mobile, regardless of whether they were assayed on infected or non-infected neurons (video S3 and data not shown). Collectively, these data show that CD8 T cells freshly purified from BDV-infected rat brains establish stable conjugates with infected neurons and that this interaction depends upon both neuronal infection and MHC I expression.

Intriguingly, a fraction of brain-purified CD8 T cells formed long projections once arrested on BDV-infected neurons, reminiscent of the T cell-extended processes (TCEP) recently described by McDole *et al.* [34]. These TCEP were 0.5 µm thick and 50 µm long on average, although we observed projections of up to 270 µm. Several TCEP were sometimes observed from a single CD8 T cells and displayed a very dynamic behavior (video S4). This dynamic behavior was also evidenced by their occasional rapid contraction (video S5). Such projections were never observed in CD8 T cells purified from lymph nodes.

**Figure 4:**
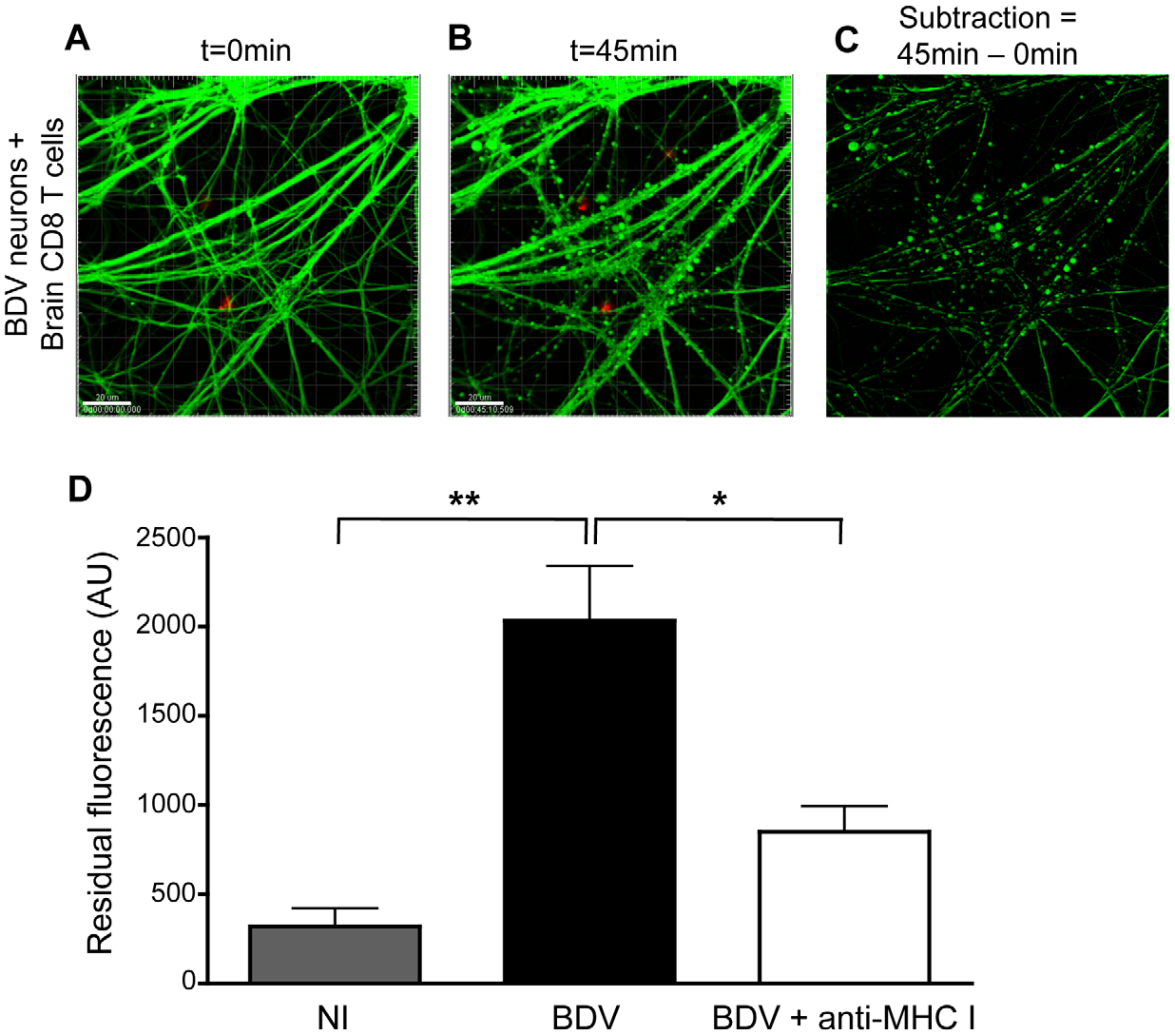
Interaction with brain-purified CD8 T cells induces early morphological changes of BDV-infected neurons. (A) A representative BDV-infected neuronal network loaded with calcein AM (green) at the start (t = 0 min) and (B) 45 min after coculture with PKH-26 stained brain-derived CD8 T cells. (C) Representative example of the image analysis performed to quantify the formation of calcein beading figures. Upon subtraction between images shown in (A) and (B), the residual fluorescence signal corresponds to calcein beading. (D) Quantification of the differences in fluorescence values, expressed as arbitrary units (AU). Values are expressed as means±sem of 5 to 6 independent experiments. *, p<0.05; **, p<0.01 by Mann-Whitney *U*-test.

Brain purified CD8 T cells induce early changes in neuronal permeability, associated with increased neuronal electrical activity. We then sought to analyze the impact of this stable interaction with CD8 T cells on BDV-infected neurons. When calcein-loaded infected neurons were incubated with brain-purified CD8 T cells, we observed the formation of “axonal beading” figures in BDV-infected neurons, revealed by the formation of calcein dots lining the neurites, while there were no visible changes in the neuronal network of control non-infected neurons incubated with brain CD8 T cells (compare Figure 4A with 4B and video S6 with video S7 and S8). Upon incubation with CD8 T cells, calcein beading figures were observed throughout the culture, affecting both neurites and neuronal somas, without any noticeable proximity with the sites of CD8 T cell interaction with neurons (video S8). In order to quantify this phenomenon, neurons were imaged for 45 minutes after addition of brain CD8 T cells and levels of calcein fluorescence measured at the beginning of the experiment were subtracted from levels obtained at the end of the incubation. The resulting differences in fluorescent signals therefore provided a good estimate of calcein beading (Figure 4C and 4D). While there were only minimal changes when brain CD8 T cells were applied to non-infected neurons, we detected significant residual fluorescence when using BDV-infected neurons. Consistent with a role of CD8-neuron cognate interaction in this process, incubation of BDV-infected neurons with anti-MHC I antibody markedly decreased calcein beading (Figure 4D).

**Figure 5:**
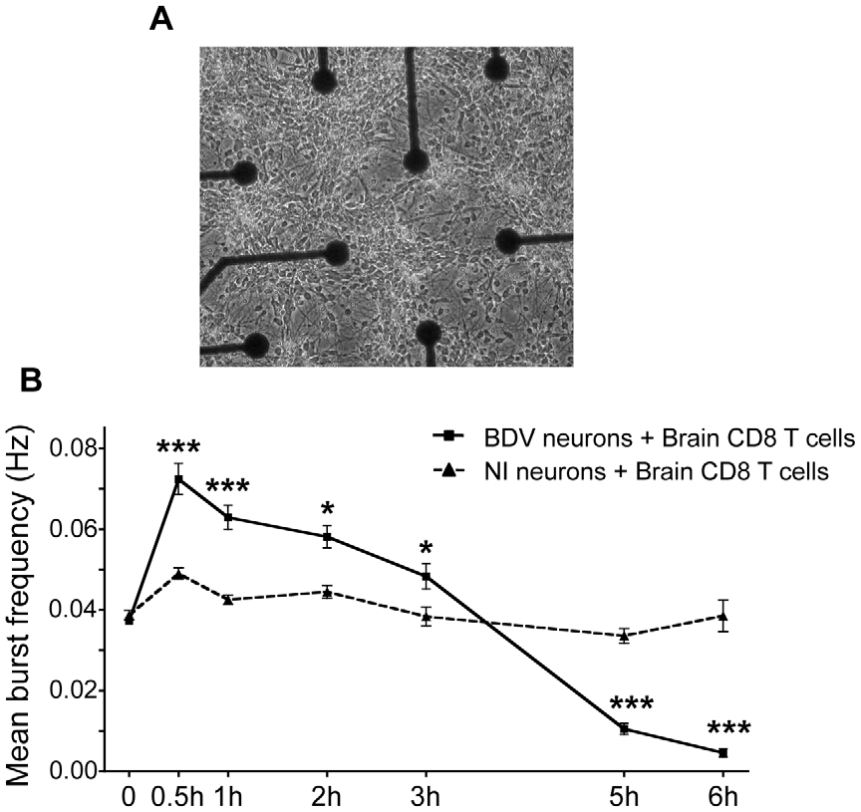
Analysis of electrical properties of neurons upon contact with brain CD8 T cells. (A) Representative view of cortical neurons cultured in an MEA dish. Electrodes are spaced by 200 µm; electrode diameter is 30 µm. Original magnification, X50. (B) Quantitative analysis of the mean burst frequency for non-infected (NI) neurons and BDV-infected neurons upon coculture with brain CD8 T cells (at a ratio of 1∶1). The mean burst frequency was first calculated under spontaneous conditions and at various times after the beginning of coculture. For each condition, data were acquired from 100 to 460 electrodes, from five independent experiments. Values are expressed as means ± sem *, p<0.05; ***, p<0.001 by unpaired Student's *t*-test.

Given the changes in neuronal morphology consecutive to incubation with brain CD8 T cells, we next studied its impact on the electrophysiological properties of neuronal networks. We used a system based on microelectrode arrays (MEA), which allows to monitor the firing pattern of a neuronal network grown on a grid of sixty electrodes embedded in a culture dish (Figure 5A) [35]. We assessed the impact of incubation with CD8 T cells by measuring the frequency of grouped action potentials, or bursts, over the neuronal network (Figure 5B). Before adding CD8 T cells, spontaneous firing frequencies were similar whether neurons were infected or not with BDV (0.037±0.0008 Hz vs. 0.038±0.0011 Hz), in agreement with our previous studies [28], [30]. Upon addition of brain-purified CD8 T cells (at a ratio of 1 to 1), the electrophysiological properties of non-infected neurons remained remarkably stable throughout the whole experiment (0.038±0.0038 Hz after 6 h of incubation). In sharp contrast, the addition of CD8 T cells to BDV-infected neurons triggered a significant increase of the mean burst frequency, which doubled to attain 0.074±0.0038 Hz. Remarkably, the neuronal network remained electrically active up to 3 h after addition of CD8 T cells. Thereafter, we witnessed a decline of the firing pattern, with neurons becoming nearly completely silent by 6 h of incubation (Figure 5B).

### Longer incubation times with brain-purified T cells lead to neuronal apoptosis

**Figure 6:**
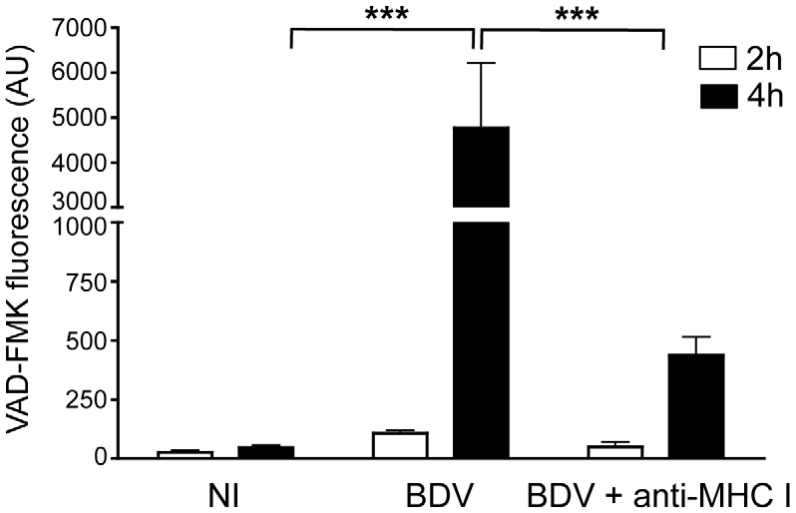
Quantitative analysis of neuronal apoptosis following incubation with brain CD8 T cells. Apoptosis was measured using the fluorescent probe FLICA, and levels of VAD-FMK fluorescence were determined on 5 randomly selected fields for each condition. This analysis was performed upon incubation for 2 hours (white bars) or 4 hours (black bars) with brain purified CD8 T cells (at a ratio of 1∶1) using non-infected neurons (NI), BDV-infected neurons, or BDV-infected neurons treated with a blocking MHC I monoclonal antibody 1 h prior to washes and addition of CD8 T cells. Values are expressed as means ± sem from 5 separate experiments. ***, p<0.001 by Mann-Whitney *U*-test.

To explore the basis for this electrical silencing occurring after longer incubation times with CD8 T cells, we analyzed whether interaction with CD8 T cells eventually triggered neuronal apoptosis. The fluorescent probe FLICA, which involves covalent binding of fluorescent Z-VAD to activated caspases, was used to detect activation of caspases 3 and 7 in live neurons. The intensity of fluorescent signals therefore provided a direct quantification of apoptosis (Figure 6). Consistent with the maintenance of their electrical activity, we did not detect significant apoptosis in non-infected neurons, even 4 h after addition of brain CD8 T cells. BDV-infected neurons did not display any caspase induction up to 2 h after incubation with CD8 T cells, but after 4 h we detected a strong increase in fluorescence, indicating prominent neuronal apoptosis. This observation was consistent with the delayed loss of electrical activity at longer time points of incubation (Figure 5B). At longer time points, we noted a progressive disaggregation of the neuronal network, which was however apparent only 6 to 8 hours after incubation with CD8 T cells (data not shown). Pre-incubation of BDV-infected neurons with anti-MHC I antibody significantly reduced neuronal apoptosis (Figure 6), further indicating that neuronal attack by CD8 T cells is a process which depends on the recognition of MHC I/viral antigen complexes at the surface of neurons.

## Discussion

Besides their central role in the response to neurotropic viral infections, CD8 T cells are increasingly being recognized as key players in the pathogenesis of many neuroinflammatory diseases, including multiple sclerosis [36]–[38]. It is thought that CTL act as effector cells and contribute to tissue damage, based on their preferential accumulation in parenchymal infiltrates. The modalities of action of CD8 T cells in the CNS and notably the interplay between immune regulation and pathogen control are, however, complex and variable. Here, we provide novel information concerning the modalities of interaction between antiviral CTL and infected neurons.

Our findings that BDV infection triggers MHC I expression by neurons were surprising. In general, viruses are better known for their capacity to down regulate or prevent the expression of MHC I molecules on the cell surface, through the expression of immunomodulatory viral proteins. Relevant examples of such proteins include HIV Nef, KSHV MIR1 and MIR2, myxoma virus MV-Lap or several CMV proteins [39]–[41]. These mechanisms of control should theoretically be even tighter in neurons, which are less prone to express MHC I molecules. Nevertheless, in the case of Flaviviruses such as West-Nile virus, it has been previously shown that CD8 T cells could recognize and destroy infected neurons, although expression of MHC I molecules was not formally assessed [42], [43]. There may be several non-exclusive mechanisms to explain our findings. First, as impairment of neuronal electrical activity has been shown to induce MHC I expression, one hypothesis is that BDV infection may trigger MHC I expression through its effects on neuronal activity. Indeed, we have previously shown that BDV can impair synaptic plasticity [28], [30] and these electrophysiological alterations could play a role in MHC I induction. Alternatively, BDV infection of neurons could trigger the production of type-1 interferons, which in turn, could induce MHC I expression. Recent evidence has clearly demonstrated that neurons can take part to the antiviral defense by being both IFN-α/ß producers and responders [44]. In addition, it is now well established that IFN-α/ß can induce detectable levels of MHC I expression by neurons, although not as efficiently as IFN-γ [1]. The fact that levels of MHC I expression induced by BDV were similar to those obtained upon exogenous application of IFN-ß (Figure 1D and 1E) provides indirect evidence in support of this hypothesis. Also consistent with these findings, a microarray analysis provided experimental evidence of a type-1 interferon signature in BDV-infected neurons (Table S1). We also confirmed our microarray data by demonstrating the up regulation of interferon-stimulated genes in BDV-infected neurons, by using both real-time quantitative RT-PCR and western blot analyses (Figure S2). Given that our neuronal cultures were prepared from embryonic (E18) cortical tissue and further cultured for a few weeks, we cannot exclude the possibility that our neurons may behave differently than fully mature neurons, in particular regarding their MHC I inducibility.

Even more surprising were our findings that neurons could not only express MHC I but also present viral antigens CD8 T cells, triggering the secretion of cytokines such as IFN-γ and IL-10. These findings uncover a novel aspect of neuro-immune interactions and reveal that neurons can process and present antigenic peptides to CTL, an aspect which could not be assessed in experimental systems using peptide-pulsed neurons. This is also consistent with previous reports showing that neurons express different components of the molecular machinery required for epitope processing and MHC I presentation, such as TAP1/TAP2 and LMP2/LMP7 [45], [46]. Based on our cytokine secretion data and imaging studies, antigen processing and MHC I presentation by neurons are clearly functionally relevant. Indeed, CTL arrest and the ensuing changes in neuronal permeability and apoptosis were all significantly prevented or delayed by the addition of an antibody masking MHC I molecules. Interestingly, brain-infiltrating CD8 T cells produced the cytokine IL-10 in addition to IFN-γ at the peak of BDV-induced encephalitis. Similar observations have been made recently in a Coronavirus-induced encephalitis model [47]. This secretion of IL-10 has been interpreted as a mechanism protecting critical organs from bystander tissue injury, while permitting viral clearance [47], [48].

Upon CTL arrest, we observed the formation of highly dynamic structures, designated as TCEP in a recent report studying CD8 T cells specific for Theiler's virus [34]. It was suggested that these dynamic cellular projections could confer a higher motility to lymphocytes, allowing them to navigate through brain tissue or sample the microenvironment surrounding the cell. Since such TCEP have now been described in two separate models of neurotropic virus infections, it would be important to assess in future studies whether these modifications of lymphocyte morphology are organ-specific and characteristic of lymphocytes infiltrating the CNS, or if they are dependent on their engagement of infected cells within this tissue.

Another interesting aspect concerned the modalities of attack of CTL against BDV-infected neurons studied by real-time imaging. Within 45 minutes after CTL addition, we observed changes in neuronal membrane permeability, revealed by the formation of calcein dots lining the neurons. These were accompanied by morphological changes, variably designated as axonal beading or blebbing by several authors, which appear to represent a general response of neurons to various stressing insults. In particular, they have been visualized in neurons following axonal transection and Wallerian degeneration [49], oxidative stress [50], ischemia [51], trauma [52] or even allogeneic CTL [53]. Although the underlying mechanism is not well understood, it seems to be accompanied by mitochondrial dysfunction and delayed cell death. Recently, it was suggested that calcein leakage from neurons following oxygen-glucose deprivation could result from opening of neuronal hemichannels, a type of half-gap junctions that form large-conductance channels and allow flux of ions and molecules [51]. It will be interesting to test whether this is also the case following CD8 T cell engagement.

Remarkably, despite these early changes in neuronal permeability, the electrical properties of infected neurons were preserved for a significant duration after addition of brain-purified CTL. This contrasted with the immediate shutdown of electrical signaling, both in single neurons and networks, which follows incubation of OVA peptide-pulsed neurons with primed OT-I cells, as recently described by Meuth et al. [15]. Since neurons were killed rapidly upon recognition by highly differentiated CD8 OT-I cells, our observations suggest that impairment of electrical activity upon CTL encounter may also be dependent on the intensity of the “lethal hit” delivered by the CTL. Indeed, the different kinetics of CTL engagement with neurons in our system is probably related to the fact that CD8 T cells purified for the brains of BDV-infected rats were not subjected to further in vitro stimulation and that the neurons were undergoing a natural viral infection, with the density of MHC I / cognate peptides being closer to physiological levels.

At present, we can only speculate about the mechanisms of neuronal death triggered by CTL. CD8 T cells purified from brains of BDV-infected rats expressed all cytolytic effectors, including IFN-γ, perforin, granzyme-B or FasL. Both Fas/FasL and perforin/granzyme pathways have been demonstrated to be effective against neurons upon engagement with CTL [14], [54], [55]. Since cell death appears to develop with slower kinetics when triggered through the Fas/FasL pathway [56], [57], given our observations that BDV-infected neurons are still electrically active after three hours, we would favor the hypothesis that killing of BDV-infected neurons preferentially occurs by this route. However, previous studies in the rat model of BDV infection have also shown expression of perforin mRNA in the brain [24]. Alternatively, the relatively low density of MHC I expression triggered by infection, together with a restricted expression of perforin could lead to a limited release of cytolytic granules towards the infected neurons. Finally, protection against excessive apoptosis may also be conferred by BDV infection per se. Recently, it was shown by Poenisch et al. that the BDV X protein could protect cells against Fas-mediated apoptosis in vitro [58]. In our system, however, the kinetics or characteristics of CTL-induced apoptosis were unchanged when we used neurons infected with the BDV-X(A6A7) mutant [58], which has lost its capacity to block Fas-induced apoptosis in vitro, resulting from loss of the mitochondrial localization of the X protein (Figure S3).

Our findings have several important implications. First, we confirmed the capacity of neurons to process and viral antigens during the course of a natural infection, leading to simulation of CD8 T cells and production of inflammatory cytokines. Therefore, beyond being the mere targets of CD8-mediated cytotoxicity, neurons may play an active part in the development of CNS inflammation through T cell activation and cytokine release. Second, the slow kinetics of neuronal death suggests that this progressive neuronal loss of function may leave open a window of opportunity for future treatments. Very recently, it was shown that the immune-mediated axonal damage that develops during autoimmune encephalomyelitis (in mice) or multiple sclerosis (in humans) was a slow and likely reversible process [59]. These authors described this process as “focal axonal degeneration”, beginning with axonal swelling and mitochondrial dysfunction, very reminiscent of the axonal beading phenomenon described herein. A better understanding of the mechanisms involved in our system may provide novel putative targets that could then be tested in other systems triggering axonal death, be they virally-mediated or not.

## Materials and Methods

### Ethics statement

This study was carried out in strict accordance with EU regulations and with the recommendations of the French national chart for ethics of animal experiments (articles R 214- 87 to 90 of the “Code rural”). The protocol was approved by the committee on the ethics of animal experiments of the région Midi Pyrénées and by IFR 150 (permit numbers: 04-U563-DG-06 and MP/18/26/04/04). All procedures were performed under deep anesthesia as described below and all efforts were made to minimize suffering.

### Primary culture of neurons and viral infection

Primary cortical neurons were prepared from Lewis rat embryos at gestational day 18 using a previously described procedure [28], [29] with the following modifications: after dissection, the cortex tissue was dissociated by incubation for 15 min at 37°C in phosphate buffer saline (PBS) containing 10 U/ml Papain (Worthington), followed by gentle dissociation in PBS containing 1.5 mg/ml BSA and 1.5 mg/ml Trypsin inhibitor (from chicken egg, Sigma). Cultures were maintained in serum-free Neurobasal medium (Invitrogen) supplemented with 0.5 mM glutamine, 1% penicillin/streptomycin and 2% B-27 supplement (Invitrogen). Plating was performed on variable supports depending of the use (Lab-Tek chambered coverglass, Nunc, culture dishes or glass coverslips), all previously coated with poly-ornithine (Sigma) and Laminin (Roche). One day after plating, half of the cultures were infected with cell-free BDV (Giessen strain He/80). Cell-released virus stocks were prepared as described [60], [61], using persistently infected Vero cells. By 14 days post-infection, BDV infection of neurons was verified by immunofluorescence for each experiment using an anti-BDV nucleoprotein serum.

### Infection of rats

Female Lewis rats (4-week-old) were obtainedfrom Janvier SAS (Le Genest St Isle, France) and maintained in our animal facility. The day of infection, rats were anesthetized with a mixture of ketamine (50 mg/kg of body weight) and xylazine (15 mg/kg of body weight). They were inoculated intracranially with 50 µl of a 20% (w/v) stock of BDV-infected rat brain homogenate, corresponding to 1000 focus-forming units of BDV. We used the fifth brain passage in newborn rats of the Giessen strain He/80. To minimize reflux along the injection tract, the needle was left in place for 20 seconds before being slowly withdrawn.

### Isolation of brain mononuclear cells (BMCs)

BMCs were isolated by a method adapted from previously described procedures [24], [32], [62]. Briefly, rats were deeply anesthetized and perfused with 50 ml of PBS through the left ventricle to remove blood from the organs. Brains were collected in Hank's buffered salt solution (HBSS) containing 20 mM Hepes (HH) and dissociated using a glass Potter. Brain suspensions were enzymatically digested for 1 hour at 37°C in HH containing collagenase D (1 mg/ml), trypsin inhibitor (TLCK, 0.5 µg/ml) and DNase I (10 µg/ml). The digested suspensions were filtered (70 µm cell strainer, Falcon), pelleted and resuspended in 30 ml of 30% Percoll (Pharmacia). This solution was carefully placed on top of 10 ml of 70% Percoll and centrifuged for 30 min at 2000 rpm at 20°C. BMCs were collected at the interface between the 30% and 70% Percoll layers, extensively washed with RPMI 1640 medium (Invitrogen) and directly used for FACS staining and cell sorting. Cell suspensions were also prepared from the cervival lymph nodes of the same animals, filtered as above, washed with PBS containing 5% fetal calf serum (FCS) and kept on ice until FACS staining and cell sorting.

### Flow cytometry and cell sorting

The monoclonal antibodies (mAbs) used were as follows: PE-conjugated anti-rat TCRαβ (clone R73) (PharMingen, San Diego, CA); anti-rat CD8β (clone 3.4.1) either FITC-conjugated (PharMingen, San Diego, CA) or Alexa 647-conjugated (Biolegend). For staining, BMCs or lymph node cells (LNC) were suspended in FACS buffer (consisting of PBS with 5% FCS) containing the different mAbs and incubated for 20 min at 4°C. After extensive washes with FACS buffer, CD8 T cells were sorted based on PE^+^ and (FITC^high^ or Alexa-647^high^) expression using a FACS Aria II-Sorp (BD Biosciences). The purity of the cells was always higher than 99%. Flow cytometry data were collected on FACSCalibur or LSRII cytometers (BD Biosciences) and analyzed using FlowJo software (TreeStar, version 8.8.6).

### Assessment of MHC I expression on neurons by flow cytometry

Neurons grown on 35 mm dishes were washed with Tyrode's solution (119 mM NaCl, 5 mM KCl, 2 mM CaCl_2_, 2 mM MgCl_2_, 25 mM Hepes, 30 mM glucose) prior to incubation at 4°C for 45 min with 5 µg/ml of purified anti-rat MHC I antibody (RT1-A, clone OX18), under gentle agitation. After several washes with Tyrode's solution, neurons were rapidly detached from the dish using 0.5% Trypsin, washed with PBS and blocked with PBS containing 2.5% FCS and 5 mM EDTA. The collected neurons were fixed and permeabilized using Cytofix/Cytoperm and Perm/Wash buffers (Becton Dickinson), according to the manufacturer's instructions and processed for staining at room temperature for 1 hour with anti-Tau antibody (Sigma-Aldrich). Neurons were washed twice in Perm/Wash buffer before FACS analysis. To induce MHC I expression, non-infected neurons were treated with either 100 U/ml mouse IFN-γ (Biosource) plus 1 µM Tetrodotoxin (TTX, Sigma), or 100 U/ml rat IFN-ß (PBL interferon source). Reagents were added to the culture medium 48 hrs before analysis of MHC I expression as described above.

### Analysis of cytokine production

IFN-γ levels were assessed by intra-cytoplasmic staining, essentially as described [63]. Briefly, BMCs or LNC were stimulated by coculture with irradiated Lewis fibroblasts (a gift from Pr. L. Stitz, Tübingen, Germany), either infected or not with BDV, in complete RPMI 1640 medium containing 10% FCS, 1% sodium pyruvate, 1% non essential amino acids, 1% L-glutamine, 1% penicillin-streptomycin, 2×10^−5^ M 2-mercaptoethanol, and 50 U/ml recombinant human IL-2 (AbCys). After 24 hours, GolgiPlug (1 µl/ml, BD Biosciences) was added in the medium and cultures were further incubated overnight. Thereafter, cells were labeled for membrane expression of TCR and CD8ß and for intra-cytoplasmic production of IFN-γ using DB1-FITC mAb (a gift from Dr. A. Saoudi, Toulouse, France) and analyzed by flow cytometry.

Cytokine levels were also measured in the supernatants as follows: directly after cell sorting, CD8 T cells purified from the brain or lymph nodes (1.5×10^5^ cells/well) were incubated with BDV-infected or non-infected neurons grown in 96-well culture plates (Falcon, Becton Dickinson). Forty-eight hours after coculture, cytokine production (IL-4, IL-5, IL-6, IL-9, IL-10, IL-13, IL-17, IFN-γ and TNF-α) was examined in the cell supernatants by Luminex multiplex immunoassay (Millipore).

### Immunofluorescence

Standard immunofluorescence was performed as described previously [29]. Briefly, neurons grown on glass coverslips were fixed for 20 min at room temperature with 4% paraformaldehyde, permeabilized using PBS + 0.1% Triton-X100 during 4 min, rinsed with PBS, and blocked overnight at 4°C with PBS + 2% normal goat serum. Incubation for 1 h at room temperature or overnight at 4°C with primary antibodies was followed, after several washes in PBS, by a 1-h incubation at room temperature with secondary antibodies. After extensive washing, coverslips were mounted using ProLong Gold (Molecular Probes).

### RNA preparation and analysis

Total RNA was isolated from highly purified CD8 T cells using the RNeasy Minikit (Qiagen, Courtaboeuf, France). Reverse transcription was performed with 10 µL of total RNA (5 to 10 ng), random hexamer primers (0.1 µg) (Gibco BRL), and SuperScript Reverse Transcriptase (200 U; Gibco BRL). cDNAs were stored at −20°C until use.

Quantitative cDNA amplification was performed according to manufacturer's instructions. The products of polymerase chain reaction (PCR) LightCycler amplification were detected with SYBR green I dye (Roche Diagnostics). PCR cycling conditions were 50°C for 2 minutes and 95°C for 10 minutes, followed by 40 cycles of 95°C for 15 seconds, 60°C for 1 minute, followed by the final melting curve program. The melting curve analysis of PCR products together with their analysis by electrophoresis revealed the presence of a single amplicon at the expected size. Each sample was run in duplicate and mean values were used for quantitation.

Relative quantification of mRNA concentrations was performed with the standard curve method, with amplification of target mRNA and actin mRNA for normalization. The relative amount of mRNA in each sample was calculated as the ratio between the target mRNA and the corresponding endogenous control actin mRNA.

The primers used were as follows. IFN-γ: 5′-GCCAAGTTCGAGGTGAACAAC-3′, 5′-TTCATTGACAGCTTTGTGCTGG-3′; FasL: 5′-AAAAGCAAATAGCCAACCCCAG-3′, 5′-AGCCTCATTGATCACAAGGCC-3′; GranzymeB: 5′-GACAGATGGCAGCAACTGAA-3′, 5′-GGCAGAAGCATTCCATTCAT-3′; Perforin: 5′-GGAAGCAAACGTGCATGTGT-3′, 5′-GCGAAAACTGTACATGCGACA-3′; β-actin 5′-TGGAATCCTGTGGCATCCATGAAA-C-3′, 5′-TAAAACGCAGCTCAGTAACAGTCCG-3′.

### Labeling of cells for imaging

After cell sorting to high purity (>99%), CD8 T cells were labeled with PKH-26 red fluorescent cell linker kit (Sigma), according to the manufacturer's instructions. Briefly, cells were stained with 4 µM PKH-26 at room temperature for 5 min, the reaction was stopped by adding FCS and cells were washed once in complete RPMI medium containing 10% FCS. In parallel, neurons grown on Labtek chambered coverglass were labeled with 1 µM calcein-AM (Molecular Probes) in neuronal medium for 30 min at 37°C. Neurons were then washed twice with Tyrode's solution.

### Time-lapse confocal microscopy

Fluorescence measurements and imaging were performed on a Zeiss LSM-510 inverted confocal microscope with 10X, 20X or 40X objectives, whilst maintaining the cells at 37°C and 5% CO2. To minimize photobleaching, one frame was captured every 20 s on average. Single-cell-tracking analysis was performed automatically (intensity >130±20; size ≈10 µm) with Imaris sotware and only tracks with durations of >120 s were included in the analysis. Image sequences of the time-lapse recordings were processed using Metamorph and Imaris softwares and 3-D images were generated with Imaris.

### Quantification of neuronal beading

For each movie, the first picture in the green channel (corresponding to neuronal staining) was subtracted from the last one (45 min later) in order to obtain the difference in green fluorescence due to the formation of beads between these two time points.

### Assessment of neuronal apoptosis

After coculture with CD8 T cells, neurons were washed with neuronal culture medium and labeled with Image-iT LIVE Green Caspase Detection Kit (Molecular Probes) which provides FLICA reagent specific for caspase-3 and -7. Staining was performed according to the manufacturer's recommandations. At different times after incubation, levels of fluorescence intensities were measured on microsopic fields chosen at random.

### Electrophysiology

Neuronal cortical cultures were prepared from Lewis rats as described above and seeded at a density of 10^5^ cells on multi-electrode arrays (MEA, Multi Channel Systems GmbH, Reutlingen, Germany). Half of the MEA dishes were infected with BDV on day 1. After addition of highly purified CD8 T cells (at a ratio of one CD8 T cell per neuron), signals corresponding to the electrical activity from the 60 electrodes of the MEA were recorded using the MC Rack Software (Multi Channel Systems GmbH, Reutlingen, Germany), which allows both online visualization and raw data storage. The signal corresponding to the firing of a single action potential by a neuron in the vicinity of an electrode was identified as a spike. We also detected high frequency grouped spikes trains, known as bursts, which represent an important parameter of the analysis of neuronal network activity [64]. Bursts were defined as a series of more than 3 spikes occurring in less than 100 ms. Spikes and bursts were detected by a dedicated analysis software developed at INSERM U862 (Bordeaux, France) [35], which computes the signal obtained from the electrodes, calculates a threshold and detects a spike every time the signal crosses this threshold with a negative slope. The threshold was set to a minimum of three standard deviations of the average noise amplitude computed over the whole recording and applied from the signal averaged value as a baseline [65]. For each time point, recordings were performed over a 3 min period, and the mean burst frequency was calculated by averaging the results obtained for all electrodes.

### Data analysis

Comparisons between groups were performed with different statistical tests (Student's *t*-test, Mann-Whitney *U*-test or Kruskal-Wallis) using the GraphPad Prism software.

## Supporting Information

Figure S1 Analysis of cytokine levels for IL-6, IL-17 and TNF-α in supernatants of cultures of CD8 T cells with neurons, either infected with BDV or not. Supernatants were assayed after 48 hours of culture using Luminex multiplex kits. Levels of IL-4, IL-5, IL-9, IL-13 were below the detection threshold of our assay (4.88 pg/ml). Values are expressed as mean concentrations ± sem of four independent experiments.
(TIFF)

Figure S2 Analysis of expression of interferon-related genes in BDV-infected neurons. (A) Determination by real-time quantitative RT-PCR of the relative mRNA levels of genes listed at the bottom of the graph in BDV-infected neurons compared to non-infected ones. Values are normalized for GAPDH mRNA levels. Values are expressed as means ± sem of three independent experiments. Primer sequences used for RT-PCR are available on request. (B) Western blot analysis. Equivalent protein amounts of non-infected (NI) or BDV-infected neurons were analyzed by western-blot with specific antibodies for STAT-1 and Mx1. Expression of GAPDH was used to normalize expression. Peroxidase activity was revealed using the Supersignal West Pico Chemiluminescent substrate (Pierce) and quantification was performed by densitometry using Scion Image software (Scion Corporation). Bottom graph shows the quantification of three independent experiments. Quantification results are expressed as percentage of increase relative to NI neurons. Values are expressed as means ± sem.
(TIFF)

Figure S3 Neurons infected with the BDV-X(A6A7) mutant do not exhibit significant change in the susceptibility to apoptosis triggered by CD8 T cells. Neuronal apoptosis was quantified as described in Figure 6, following incubation for 4 hours with brain CD8 T cells (at a ratio of 1∶1). Levels of VAD-FMK fluorescence were determined on 5 randomly selected fields for each condition. This analysis was performed upon incubation of brain purified CD8 T cells with non-infected neurons, neurons infected with wild-type BDV or the BDV-X(A6A7) mutant, as well as BDV-infected neurons treated with a neutralizing MHC I monoclonal antibody 1 h prior to washes and addition of CD8 T cells. One representative experiment out of two is shown.
(TIFF)

Table S1 Genes most highly induced in BDV-infected neurons as determined by microarray analysis. The table shows only the genes for which the difference between control (non-infected) and BDV-infected neurons was of more that 2-fold. Genes indicated in bold characters correspond to genes related to the interferon pathway. FC: fold change; N/A not available. For microarray analysis, total RNA was extracted from BDV-infected and non-infected neurons after 13 days in culture using an RNeasy mini kit as described in the materials and methods. Triplicate samples were obtained for each condition. RNA quality was verified using nanochips on an Agilent 2100 bioanalyzer (Agilent). Gene expression profiles were analyzed using Affymetrix microarrays for the rat genome (RAE230 v2.0). Analysis was performed using the Affymetrix platforms of “Génopôle Ile de France” for RNA labeling and hybridization. Statistical analysis was performed with the Toulouse Genopole (http://genopole-toulouse.prd.fr/).
(DOC)

Video S1 Representative example of the behavior of brain-purified CD8 T cells incubated with non-infected neurons. CD8 T cells purified from the brains of BDV infected rats were labeled with PKH-26 (red) and added to calcein-loaded, non-infected neurons. Movie is based on one image capture every 20 s using a 10X objective. Trajectories of CD8 T cells are depicted as color-coded tracks to represent increasing time, as described in the legend of figure 3. Time counter and scale bar are inserted at the bottom left of the video. Note the highly mobile and scanning behavior of CD8 T cells.
(MP4)

Video S2 Representative example of the behavior of brain-purified CD8 T cells incubated with BDV-infected neurons. Movie acquisition and analysis was as described for video S1. Note the reduced mobility of CD8 T cells in contact with BDV-infected neurons.
(MP4)

Video S3 Representative example of the behavior of CD8 T cells purified from the cervical lymph nodes of a BDV infected rat and incubated with BDV-infected neurons. Movie acquisition and analysis was as described for video S1. Lymph node purified CD8 T cells are very mobile, even in contact with BDV-infected neurons.
(MP4)

Video S4 Morphological features of a brain-purified CD8 T cell in contact with BDV-infected neurons. CD8 T cells were labeled with PKH-26 (red) and added to neurons as described for video S1. This video shows a closer view of one CD8 T cell to illustrate the formation of T cell extended projections (TCEP) displaying a dynamic behavior. The focus of the microscope was set on the CD8 T cell to visualize the projections and the neuronal network is consequently less visible. Time counter and scale bar are inserted at the bottom left of the video.
(MP4)

Video S5 Projections of brain-isolated CD8 T cells are very dynamic and can rapidly retract. PKH-26-labeled CD8 T cells isolated from the brain were incubated with BDV-infected neurons. The video setup and analysis is similar to that described for video S4. Time counter and scale bar are inserted at the bottom left of the video. The projection of a T cell interacting with a neuronal soma, is clearly visible for >15 min and then rapidly retracts in less than 2 min.
(MP4)

Video S6 Incubation of brain-purified CD8 T cells with non-infected neurons does not impair neuronal network. PKH-26-labeled brain-derived CD8 T cells were added to calcein-loaded, non-infected neurons and examined as described in video S1. A representative sequence of >1 hr is shown (time counter and scale bar are inserted at the bottom left of the video), during which no changes resulting from CD8 T cell scanning were noted on the neuronal network.
(MP4)

Video S7 Incubation of brain-purified CD8 T cells with BDV-infected neurons triggers calcein beading. PKH-26-labeled brain-derived CD8 T cells were added to calcein-loaded, BDV-infected neurons and examined as described in video S2. In this representative sequence the networks appears normal for up to 30 min after incubation with CD8 T cells, followed by the progressive beading of neuronal calcein staining. Time counter and scale bar are inserted at the bottom left of the video.
(MP4)

Video S8 4-D reconstruction using Imaris software (38 minute-capture movie, accelerated 280 x) of the interaction between brain-derived CD8 T cells (labeled with PKH-26, red) and infected neurons (labeled with calcein-AM, green), similar to that shown on movie S7. The calcein beading figures consecutive to the interaction with CD8 T cells occur throughout the neuronal network and on cell somas, with no specific localization with the sites of CD8 T cell binding.
(MP4)
