# Supplementary figures and images for: Neurons are MHC Class I-Dependent Targets for CD8 T Cells upon Neurotropic Viral Infection
Source: PLoS Pathog. 2011 Nov 17;7(11):e1002393. doi: 10.1371/journal.ppat.1002393 (PMC3219726; doi:10.1371/journal.ppat.1002393)

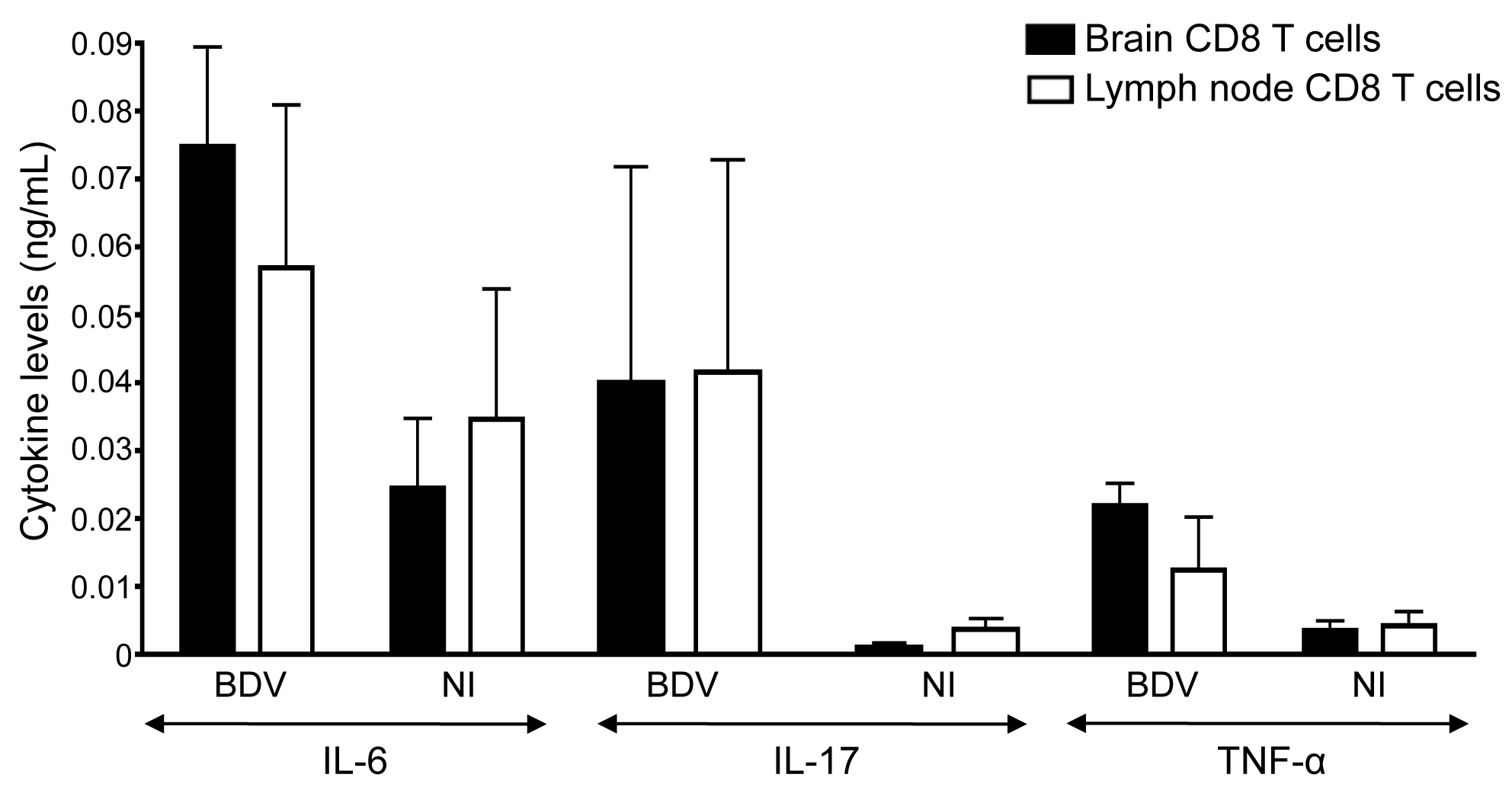

Supplement: Figure S1 — Analysis of cytokine levels for IL-6, IL-17 and TNF-α in supernatants of cultures of CD8 T cells with neurons, either infected with BDV or not. Supernatants were assayed after 48 hours of culture using Luminex multiplex kits. Levels of IL-4, IL-5, IL-9, IL-13 were below the detection threshold of our assay (4.88 pg/ml). Values are expressed as mean concentrations ± sem of four independent experiments. (TIFF) [file ppat.1002393.s001.tiff]

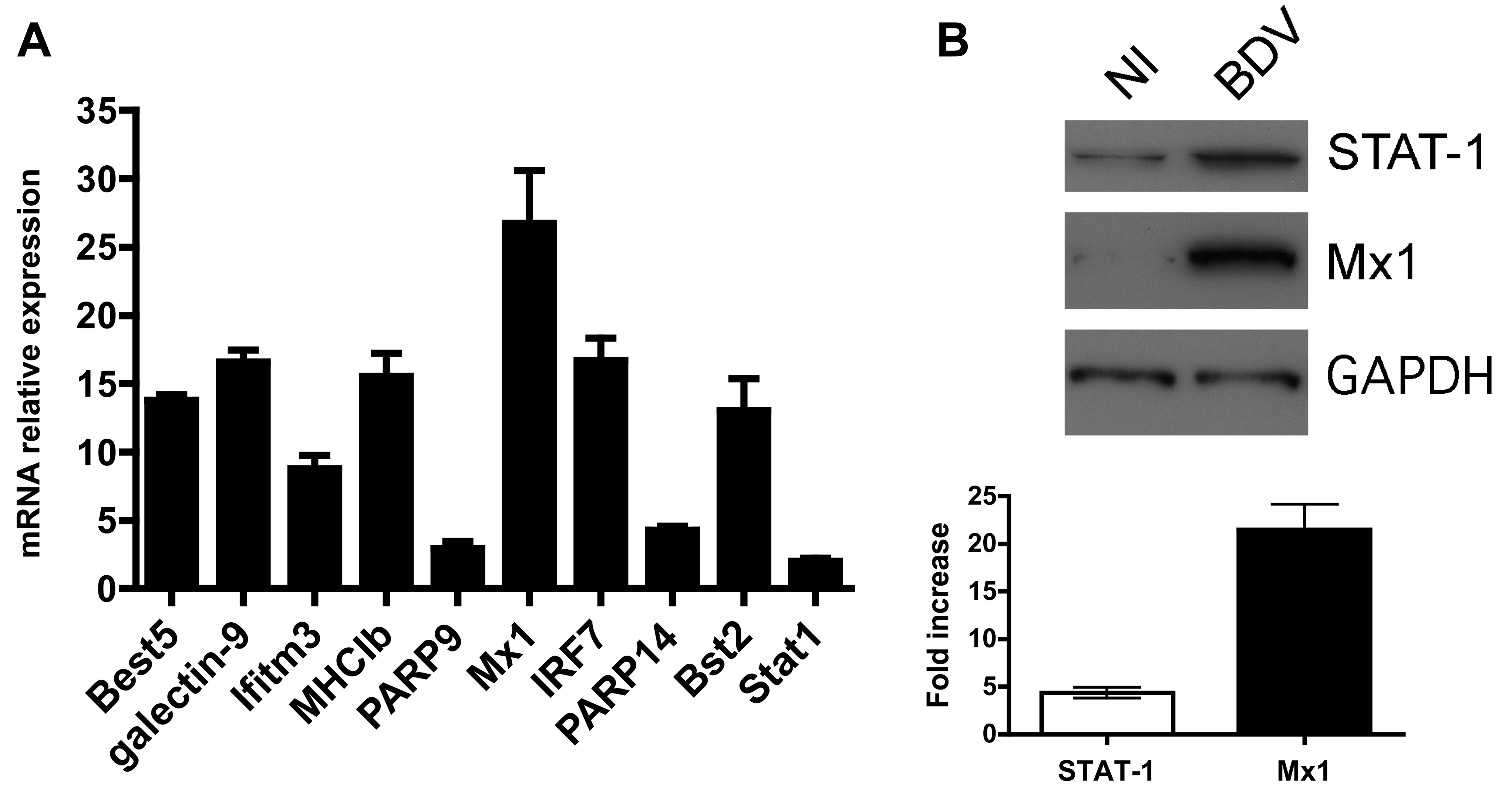

Supplement: Figure S2 — Analysis of expression of interferon-related genes in BDV-infected neurons. (A) Determination by real-time quantitative RT-PCR of the relative mRNA levels of genes listed at the bottom of the graph in BDV-infected neurons compared to non-infected ones. Values are normalized for GAPDH mRNA levels. Values are expressed as means ± sem of three independent experiments. Primer sequences used for RT-PCR are available on request. (B) Western blot analysis. Equivalent protein amounts of non-infected (NI) or BDV-infected neurons were analyzed by western-blot with specific antibodies for STAT-1 and Mx1. Expression of GAPDH was used to normalize expression. Peroxidase activity was revealed using the Supersignal West Pico Chemiluminescent substrate (Pierce) and quantification was performed by densitometry using Scion Image software (Scion Corporation). Bottom graph shows the quantification of three independent experiments. Quantification results are expressed as percentage of increase relative to NI neurons. Values are expressed as means ± sem. (TIFF) [file ppat.1002393.s002.tiff]

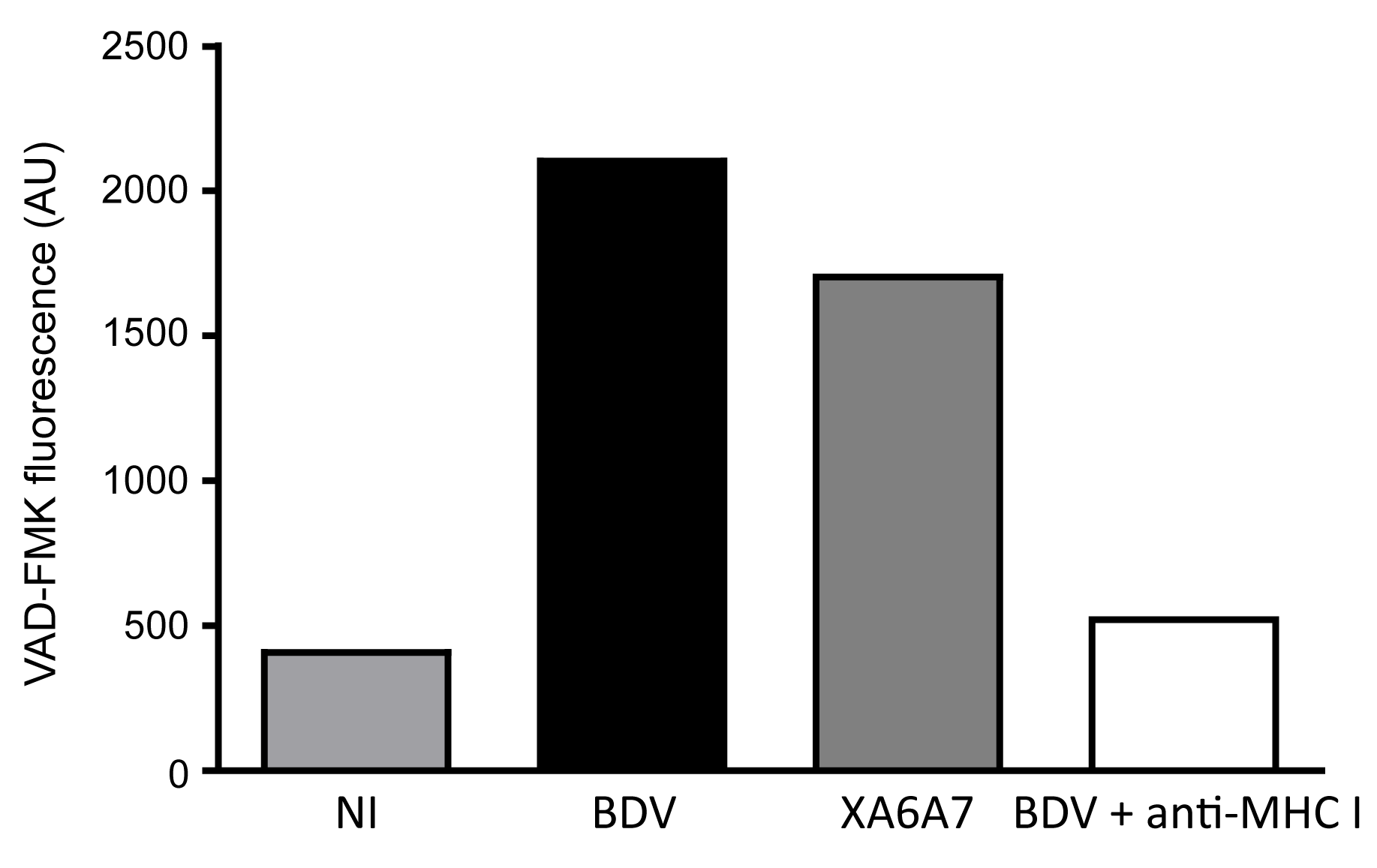

Supplement: Figure S3 — Neurons infected with the BDV-X(A6A7) mutant do not exhibit significant change in the susceptibility to apoptosis triggered by CD8 T cells. Neuronal apoptosis was quantified as described in Figure 6, following incubation for 4 hours with brain CD8 T cells (at a ratio of 1∶1). Levels of VAD-FMK fluorescence were determined on 5 randomly selected fields for each condition. This analysis was performed upon incubation of brain purified CD8 T cells with non-infected neurons, neurons infected with wild-type BDV or the BDV-X(A6A7) mutant, as well as BDV-infected neurons treated with a neutralizing MHC I monoclonal antibody 1 h prior to washes and addition of CD8 T cells. One representative experiment out of two is shown. (TIFF) [file ppat.1002393.s003.tiff]
