# Supplementary material for: Neurons are MHC Class I-Dependent Targets for CD8 T Cells upon Neurotropic Viral Infection
Source: PLoS Pathog. 2011 Nov 17;7(11):e1002393. doi: 10.1371/journal.ppat.1002393 (PMC3219726; doi:10.1371/journal.ppat.1002393)
Supplement: Table S1 — Genes most highly induced in BDV-infected neurons as determined by microarray analysis. The table shows only the genes for which the difference between control (non-infected) and BDV-infected neurons was of more that 2-fold. Genes indicated in bold characters correspond to genes related to the interferon pathway. FC: fold change; N/A not available. For microarray analysis, total RNA was extracted from BDV-infected and non-infected neurons after 13 days in culture using an RNeasy mini kit as described in the materials and methods. Triplicate samples were obtained for each condition. RNA quality was verified using nanochips on an Agilent 2100 bioanalyzer (Agilent). Gene expression profiles were analyzed using Affymetrix microarrays for the rat genome (RAE230 v2.0). Analysis was performed using the Affymetrix platforms of “Génopôle Ile de France” for RNA labeling and hybridization. Statistical analysis was performed with the Toulouse Genopole (http://genopole-toulouse.prd.fr/). (DOC) [file ppat.1002393.s004.doc]

**Supplementary Table 1**

| **Common**  **name** | Description | **FC** | **Affymetrix**  **Probe set ID** |
| --- | --- | --- | --- |
| **Best5**  **GIP-2**  **USP18**  **Gal-9**  **N/A**  **RTP4**  **Ifitm-3**  **MHCIb**  Oas1i  Oas1  **ISG12(a)**  **PARP9**  **Mx1**  Ddx58  IF-1  Ubce8  IFI644  Trim25  **N/A**  Ifih1  **ISGF3g**  **Mx2**  **IF-35**  **IRF-7**  **PARP14**  Bst2  N/A  Rpl39  **Stat1**  N/A  Unc5-B  N/A  N/A  CRBP1  HCA127  Tpcn2  TNFRSF6 | **Bone-expressed sequence tag 5 (Viperin)**  **Interferon alpha inducible protein (clone IFI-15K)**  **Ubiquitin specific protease 18 (UBP43)**  **Galectin 9 (lectin galactose binding soluble 9)**  **Interferon inducible GTPase**  **28kD interferon responsive protein**  **Interferon induced transmembrane protein 3**  MHC class Ib RT1.S3  **2'-5' oligoadenylate synthetase 1I**  **2'-5' oligoadenylate synthetase 1**  **putative ISG12(a) protein**  **poly (ADP-ribose) polymerase family member 9**  Myxovirus (influenza virus) resistance 1  DEAD (Asp-Glu-Ala-Asp) box polypeptide 58  Interferon inducible protein 1  **Ubiquitin conjugating enzyme E2L 6**  **Interferon induced protein 44**  Tripartite motif protein 25  Galectin 3 binding protein  Interferon induced with helicase C domain 1  **Interferon dependent positive acting transcription factor 3 g**  **Myxovirus (influenza virus) resistance 2**  Interferon induced protein 35  Interferon regulatory factor 7  **Poly (ADP-ribose) polymerase family, member 14**  **Bst2/DAMP-1**  flt3 ligand  Ribosomal protein L39  Signal transducer and activator of transcription 1  Interferon gamma induced GTPase  **unc-5 homolog B (netrin receptor)**  Interferon inducible GTPase 1  Transcriptional regulator protein  Cellular retinol binding protein 1  hepatocellular carcinoma-associated antigen 127  Two-pore calcium channel protein 2  Fas associated factor 1 | **18.78**  **12.47**  **11.18**  **7.97**  **7.40**  **6.33**  **6.19**  **4.75**  **4.73**  **4.34**  **4.34**  **3.99**  **3.58**  **2.98**  **2.93**  **2.87**  **2.78**  **2.66**  **2.38**  **2.33**  **2.32**  **2.32**  **2.30**  **2.30**  **2.28**  **2.24**  2.24  2.18  **2.16**  **2.11**  **2.10**  **2.09**  2.06  2.05  2.04  2.04  2.03 | 1370913_at  1382314_at  1389034_at  1387027_at  1373992_at  1379285_at  1387995_at  1388212_at  1391754_at  1371152_at  1387770_at  1376144_at  1371015_at  1391463_at  1391489_at  1373037_at  1381014_at  1374731_at  1387946_at  1385276_at  1383448_at  1387283_at  1374551_at  1383564_at  1372034_at  1390738_at  1379228_at  1367934_at  1368835_at  1396163_at  1373701_at  1377950_at  1383441_at  1367939_at  1395492_at  1383589_at  1374360_at |
